# Cystatin from Filarial Parasites Suppress the Clinical Symptoms and Pathology of Experimentally Induced Colitis in Mice by Inducing T-Regulatory Cells, B1-Cells, and Alternatively Activated Macrophages

**DOI:** 10.3390/biomedicines7040085

**Published:** 2019-10-31

**Authors:** Nalini Bisht, Vishal Khatri, Nikhil Chauhan, Ramaswamy Kalyanasundaram

**Affiliations:** Department of Biomedical Sciences, University of Illinois College of Medicine, Rockford, IL 61107, USA; nalini.bisht22@gmail.com (N.B.); vkkhatr4@uic.edu (V.K.); nikhilc3@uic.edu (N.C.)

**Keywords:** *brugia malayi* cystatin, ulcerative colitis, helminth therapy, T-regulatory cells, B-cells, DSS-induced colitis

## Abstract

Potential alternative therapeutic strategies for immune-mediated disorders are being increasingly recognized and are studied extensively. We previously reported the therapeutic potential of *Brugia malayi* derived recombinant cystatin (r*Bma*Cys) in attenuating clinical symptoms of experimental colitis. The aim of this study was to elucidate the mechanisms involved in the r*Bma*Cys-induced suppression of inflammation in the colon. Our results show that, the frequency of CD4^+^CD25^+^FoxP3^+^ regulatory T-cells was elevated in the colon and mesenteric lymph nodes. Similarly, the peritoneal macrophages recovered from the r*Bma*Cys-treated colitis mice were alternatively activated and displayed reduced expression of TNF-α and IL-6. Another finding was significant increases in IgM^+^B1a-cells in the peritoneal cavity of mice following r*Bma*Cys-treatment. These findings suggested that the regulatory cell network promoted by the r*Bma*Cys in the colon and associated lymphoid tissues is important for its anti-inflammatory activity in the dextran sulfate sodium (DSS)-induced colitis mice.

## 1. Introduction

Helminth therapy has emerged as an efficient alternative to traditional treatments against autoimmune and inflammatory disorders. Known for the polarization of immune response from Th1 to Th2 biased responses, infection with helminth parasites can suppress or prevent symptoms of autoimmune and inflammatory diseases [1,2]. However, infection with live parasites is not considered an ethically acceptable option. Therefore, much attention focused on parasite-derived excretory-secretory molecules and several studies demonstrated that the parasite-derived molecules are as efficacious as the live parasite in suppressing autoimmune and inflammatory disease conditions [3,4].

Cystatins are protease inhibitors of cysteine proteases consisting of three families; stefins (cystatins A and B), cystatins (cystatins C, E, and S), and kininogens [5]. Cystatins play a pivotal role in (i) suppressing pathogenicity in immune-mediatory disorders, (ii) modulating antigen processing and presentation by antigen presenting cells, (iii) inducing T-cell mediated immunosuppression, and (iv) inhibiting release of proinflammatory cytokines. Several pathogenic organisms including parasites are known to produce cystatins with defined immunomodulatory roles in the host [4]. These parasite-derived cystatins are well-known for their ability to trigger immunosuppressive cytokines from a number of immune cells [6]. Given its potent immunoregulatory role, cystatin of helminth parasites such as *Ascaris lumbricoides* [7], *Schistosoma japonicum, Acanthocheilonema viteae*, *Clonorchis sinensis, B. malayi* [8], and *Fasciola hepatica* has been extensively studied for its therapeutic potential in various inflammatory immune conditions including ulcerative colitis (UC) [9,10,11,12,13]. These studies showed that the parasite cystatin can reverse the inflammatory immune responses in colitis and ameliorate its clinical symptoms.

Previous studies also demonstrated that recombinant cystatin from *B. malayi* (r*Bma*Cys) can alleviate the pathology in dextran sulfate sodium (DSS)-induced colitis in a mouse model [8]. Intraperitoneal administration of r*Bma*Cys led to the reduction of the overall disease severity, decreased clinical symptoms and histopathological changes, suggesting a great potential for developing r*Bma*Cys as a therapeutic agent for colitis. Although it is clearly shown that the r*Bma*Cys treatment can downregulate inflammatory responses in the colon, the mechanism of this anti-inflammatory effect is not fully understood. Therefore, the major focus of this study was to elucidate and dissect out the potential immunological mechanism by which r*Bma*Cys induces immunosuppression and downregulation of inflammation in dextran sulfate sodium (DSS)-induced colitis in mouse.

## 2. Results

### 2.1. rBmaCys-Treatment Attenuated DSS-Induced Clinical Signs of Colitis in Mice

**Figure 1:**
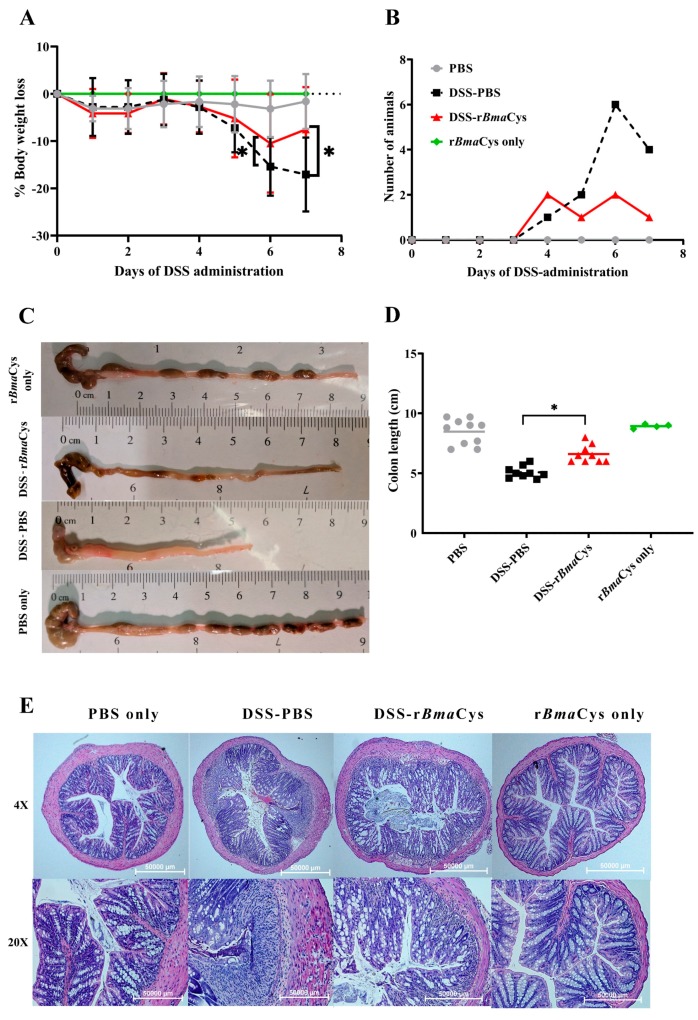
r*Bma*Cys-treatment ameliorated dextran sulfate sodium (DSS)-induced damage to the colon. Clinical signs of colitis were monitored in all the animals throughout the experimental period (day 1–7 after DSS-administration). (**A**) Loss of body weight is expressed as percentages (mean ± S.D. values). DSS-r*Bma*Cys-treated mice showed a reduced loss in their body weights compared to the DSS only group. (**B**) In the DSS-PBS group, several animals showed blood in the feces. However, by day 7, only one animal in the DSS-r*Bma*Cys treated group had blood in the feces compared to four animals in the DSS-PBS group. Thus, the clinical symptoms of colitis were substantially reduced following r*Bma*Cys-treatment. (**C**) Representative figures of colons from all the experimental groups. (**D**) The graph represents individual and means colon lengths (mean ± S.D. values) for each group of animals. r*Bma*Cys-treatment significantly reduced the damaging effects of DSS on the colon length. (**E**) H&E stained tissue sections of colon samples. Compared to the DSS-PBS group, colon tissue from DSS-r*Bma*Cys-treated mice had substantial preservation of normal colon histology and decreased cellular infiltration. * *P* ≤ 0.05 compared to DSS-PBS group as determined using one-way ANOVA followed by Tukey’s posthoc test, *n* = 10 mice per group, except the r*Bma*Cys only group, which had 4 animals.

Control mice given DSS showed progressive loss of weight reaching up to approximately a 17% loss in body weight (Figure 1A). The loss was significant from day 5. Mice treated with DSS-r*Bma*Cys also showed a slight loss in body weight initially, but by day 7 the body weight loss was less than 8% compared to the controls (*P* = 0.028). These findings suggested that r*Bma*Cys-treatment partially reversed the loss of body weight following DSS-administration. Similarly, another clinical parameter that we evaluated was hematochezia. In the DSS-r*Bma*Cys-treated group only one animal showed hematochezia on day 7 (Figure 1B), whereas, in the DSS group, four mice showed hematochezia (Figure 1B). Presence of blood in the stools suggests potential damage to the colon mucosa and associated pathology. Overall, the clinical symptoms in the DSS-r*Bma*Cys-treated colitis mice were substantially ameliorated when compared to all the groups of mice that received DSS (Figure 1A,B). The control mice that received PBS alone or r*Bma*Cys alone had no clinical symptoms of colitis.

When we measured the length of colon in each mouse following autopsy, the animals in the DSS group had the shortest colon (mean length 5.09 ± 0.5 cm), which was significantly (*P <* 0.0001) smaller compared to the PBS control group (mean length 8.47 ± 1.07 cm) (Figure 1C,D). However, in DSS-r*Bma*Cys-treated mice there was significant (*P* = 0.0005) preservation of colon (mean length 6.77± 0.86 cm) compared to the DSS group of mice (Figure 1D) and was similar to the PBS control group.

Histopathological examination of the colon sections from DSS alone group showed severe infiltration of inflammatory cells extending to the submucosa with severe damage to surface epithelium and crypt loss (Figure 1E). There was significant edema and accumulation of erythrocytes in the mucosa suggesting frank hemorrhage and disruption of the normal architecture of the colon tissue (Figure 1E). Histopathological analysis of the colon tissue from DSS-r*Bma*Cys-treated group revealed a dramatic reduction in the histopathological changes with very little or no indications of hemorrhage or mucosal edema (Figure 1E). The architecture of the colon tissue was preserved to nearly normal. Sections from the PBS control and r*Bma*Cys alone control group of mice showed normal colon tissue (Figure 1E). Results from the histopathological scoring also confirmed that DSS-induced colitis mice had severe colon damage compared to the colon tissue from DSS-r*Bma*Cys-treated colitis mice (24.6% reduction in histopathological damage score with respect to DSS alone group; Table 1).

### 2.2. rBmaCys-Treatment Significantly Decreased the Expression of Pro-Inflammatory Cytokines in the Colon Tissues

**Figure 2:**
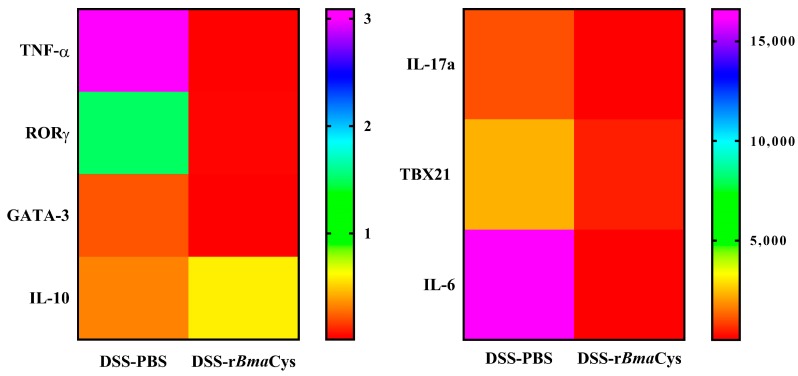
Expression of inflammatory genes and transcription factors was downregulated in DSS-r*Bma*Cys-treated mice. Colon tissues from mice were processed for RNA isolation followed by cDNA synthesis and TaqMan qPCR analysis. Relative gene expression is represented as a heat map. The results show downregulation in the expression of TNF-α, RORγ, GATA-3, IL-17a, TBX21, and IL-6 genes in the DSS-r*Bma*Cys-treated mice compared to the DSS-PBS group (*n* = 2; colon samples from male mice and female mice were pooled separately within each group to recover more cells for the analysis). Significance within the groups was determined using one-way ANOVA followed by Tukey’s posthoc test.

qPCR analysis revealed a significant reduction in the expression of key pro-inflammatory cytokine genes (TNF-α, IL-17a, and IL-6) in the colon tissue of DSS-r*Bma*Cys-treated mice (Figure 2). The changes were over 100-fold decrease (*P* < 0.05) in the TNF-α, IL-17a, and IL-6 levels in the colon tissue compared to the DSS alone group of mice. Expression of the transcription factors, RORγ (*P* < 0.05), and TBX21, which are specific for the proinflammatory cytokines, was also decreased in the colon tissue of DSS-r*Bma*Cys-treated mice compared to the DSS-alone group of mice (Figure 2). To our surprise, the expression levels of the transcription factor GATA3 also decreased in the DSS-r*Bma*Cys-treated mice compared to the DSS alone group of mice (Figure 2). However, there was a slight increase in the expression of the IL-10 gene in the colon tissues of DSS-r*Bma*Cys-treated mice compared to the DSS alone group of mice (Figure 2).

### 2.3. IL-10^+^ Tregulatory Cells (Tregs) were increased in the Colon of rBmaCys-Treated Colitis Mice

**Figure 3:**
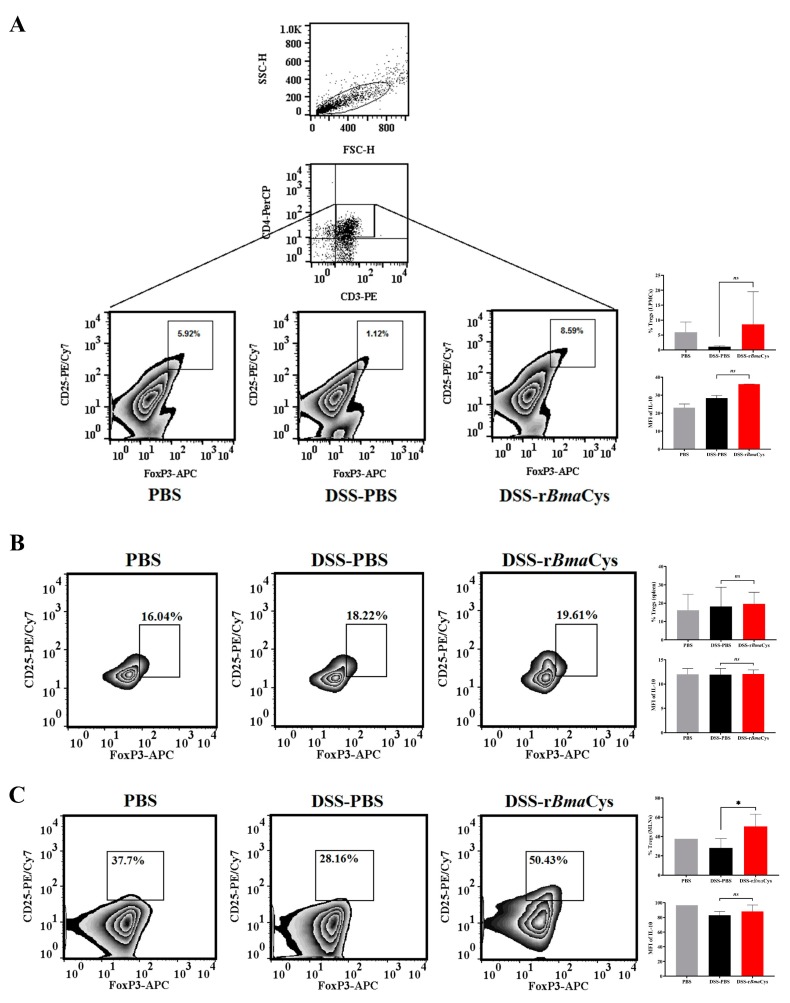
Frequency of Tregs was increased following r*Bma*Cys-treatment. (**A**) Colon tissues were homogenized and digested with collagenase, DNase, and dispase to produce a cell suspension which was then purified with percoll gradient to isolate lamina propria mononuclear cells (LPMCs). The isolated LPMCs were then subjected to flow cytometric analysis after surface staining with fluorescently labeled anti-CD3, anti-CD4, anti-CD25, and intracellular staining with anti-FoxP3 and anti-IL-10 antibodies. There was a marked increase in the percentage of Tregs in the colon LPMCs of DSS-r*Bma*Cys-treated group compared to DSS-PBS and PBS control group of mice. Intracellular staining showed that the majority of these Tregs were positive for IL-10 (mean fluorescence intensity, MFI, Σ) in the DSS-r*Bma*Cys-treated group compared to the DSS-PBS and PBS control mice. (**B**) Spleen cells were stimulated with the anti-CD3 and anti-CD28 antibodies for 72 h and then fluorescently labeled. Flow cytometric analysis showed that there was no significant difference in the percentage of Tregs or the MFI of IL-10. (**C**) However, analyses of the mesenteric lymph nodes (MLNs) from mice in each group showed that there is an increase in Tregs in the lymph nodes of DSS-r*Bma*Cys-treated mice compared to DSS-PBS mice; no difference in the MFI of IL-10 was observed between DSS-r*Bma*Cys-treated mice and DSS-PBS mice. Each bar represents mean ± S.D values. *n* = 10 mice per group, except DSS-PBS group which had 8 mice except *n* = 2 for Figure 3A (colons from 5 male mice and 5 female mice within each group were pooled to collect sufficient LPMCs for this assay). * *P* ≤ 0.05 compared to DSS-PBS group as determined using one-way ANOVA followed by Tukey’s posthoc test. *ns* = not significant.

To determine the role of Tregs in the immunomodulation caused by r*Bma*Cys treatment in DSS-induced colitis mice, we analyzed the percent frequency of CD3^+^CD4^+^CD25^+^FoxP3^+^ Tregs in the colon, spleen, and mesenteric lymph nodes (MLNs). Our results showed that the frequency of Tregs was increased in the colon lamina propria mononuclear cells (LPMCs) of DSS-r*Bma*Cys-treated colitis mice (8.59%) compared to the DSS group (1.12%) and the PBS control group (5.92%) of mice (Figure 3A). Further analysis showed a marked increase in the mean fluorescence intensity (MFI = Σ) of IL-10 in LPMCs Tregs from DSS-r*Bma*Cys-treated mice (Ʃ = 36) compared to control mice (Σ = 28; Figure 3A). Moreover, the percentage of Tregs was not significantly altered in the spleen of DSS-r*Bma*Cy*s*-treated mice (19.61%, Σ IL-10 = 12.07) compared to the controls (18.22%, Σ IL-10 = 11.94; Figure 3B). However, analysis of the MLNs showed significant (*P* ≤ 0.05) increase in the frequency of Tregs in the nodes of DSS-r*Bma*Cys-treated mice (50.43%, Σ IL-10 = 88.2) compared to the DSS alone group (28.16%, Σ IL-10 = 82.96) or the control group of mice (Figure 3C). However, these cells were not observed to be specifically secreting IL-10 (Figure 3C).

**Figure 4:**
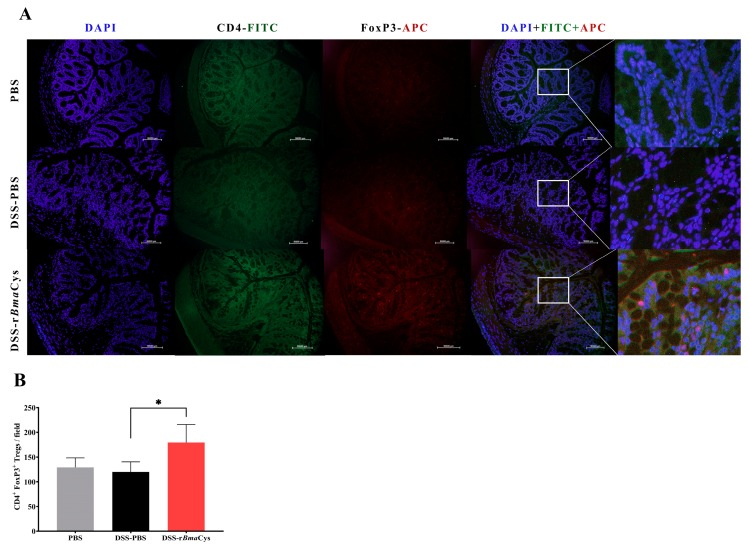
The number of Tregs were increased in the colon tissue of DSS*-*r*Bma*Cys-treated mice. Sections of the colon tissue were stained with DAPI, mouse anti-CD4 (FITC), and anti-FoxP3 (APC) antibodies. (**A**) Representative stained section from each group (200× magnification). (**B**) Total number of CD4^+^FoxP3^+^ Tregs were counted in four fields (at 200×) and are presented as mean ± S.D. number of Tregs in the colon tissue section for each group. *n* = 10 mice per group, except DSS-PBS group which had 8 mice. * *P* < 0.05 compared to DSS-PBS group as determined using one-way ANOVA followed by Tukey’s posthoc test.

Similarly, immunohistochemical studies showed a significant (*P* = 0.02) increase in the counts of CD4^+^FoxP3^+^ cells in the colonic tissues of DSS-r*Bma*Cys-treated mice (179 ± 36) compared to DSS alone group (120 ± 20; Figure 4). These results also suggest that Tregs are potentially among one of the cells that contributed to the IL-10 in the colon tissue of r*Bma*Cys-treated mice.

### 2.4. Peritoneal Macrophages from rBmaCys-Treated Colitis Mice Produced Lesser Amounts of Nitric Oxide and Th1 Cytokines

**Figure 5:**
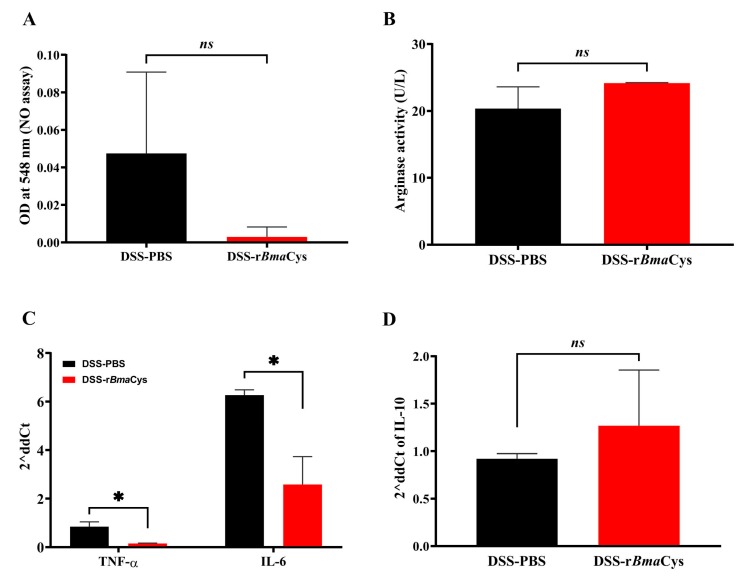
Activation markers were downregulated in the peritoneal macrophages collected from DSS-r*Bma*Cys-treated mice. Peritoneal cells from each group of mice were plated for 24 h and the culture supernatant was evaluated for levels of (**A**) nitric oxide, (**B**) arginase activity, (**C**) TNF-α and IL-6, and (**D**) IL-10. Results show that the nitric oxide levels were considerably low, but arginase 1 activity was slightly increased in the culture supernatants of plastic-adhered peritoneal macrophages collected from DSS-r*Bma*Cys-treated mice although the values were not significant compared to the DSS-PBS group. Total RNA isolated from the peritoneal macrophages was reverse transcribed to cDNA and expression levels (double delta CT value; 2^−∆∆*C*t^) of TNF-α, IL-6, and IL-10 were estimated after normalizing the values to the PBS control group. These results showed that TNF-α and IL-6 expression were significantly downregulated in r*Bma*Cys-treated mice compared to DSS-PBS control mice. The expression of IL-10 was upregulated in the peritoneal macrophages collected from DSS-r*Bma*Cys-treated mice, however was not significant compared to DSS-PBS group of mice and the PBS control mice (mRNA samples from male and female groups of mice were determined separately for qPCR assay and did not show any differences). Each bar represents mean ± S.D. * *P* ≤ 0.05 compared to DSS-PBS group determined using Students’ *t* test. *n* = 10 mice per group, except the DSS-PBS group, which had 8 mice. *ns* = not significant.

We further investigated the alternative activation of plastic-adhered peritoneal macrophages collected from DSS-r*Bma*Cys-treated mice. Nitric oxide levels were considerably decreased in the culture supernatants of peritoneal cells from DSS-r*Bma*Cys-treated mice compared to the DSS alone group and the PBS control group of mice (Figure 5A). Arginase 1 is an enzyme produced by M2 macrophages and is inversely associated with the levels of nitric oxide produced by the cells. When analyzed, the peritoneal macrophages from DSS-r*Bma*Cys-treated mice had increased arginase 1 activity (24.16 U/L) but was not significant compared to the DSS-PBS group of mice (20.35 U/L; Figure 5B). Similarly, peritoneal macrophages collected from the DSS-r*Bma*Cys-treated mice had significantly (*P* < 0.05) reduced expression levels (double delta CT value; 2δCt) of the Th1 cytokines (TNF-α; 2δCt 0.15 and IL-6; 2δCt 2.58) compared to the DSS-PBS control group of mice (TNF-α; 2δCt 0.84, IL-6; 2δCt 6.26, IL-10; 2δCt 0.92, Figure 5C). Levels of IL-10 showed some increase (2δCt 1.26) but not significant when compared to PBS controls (Figure 5D).

### 2.5. IgM^+^B1a Cells were increased in the Peritoneal Cavity of rBmaCys-Treated Colitis Mice

**Figure 6:**
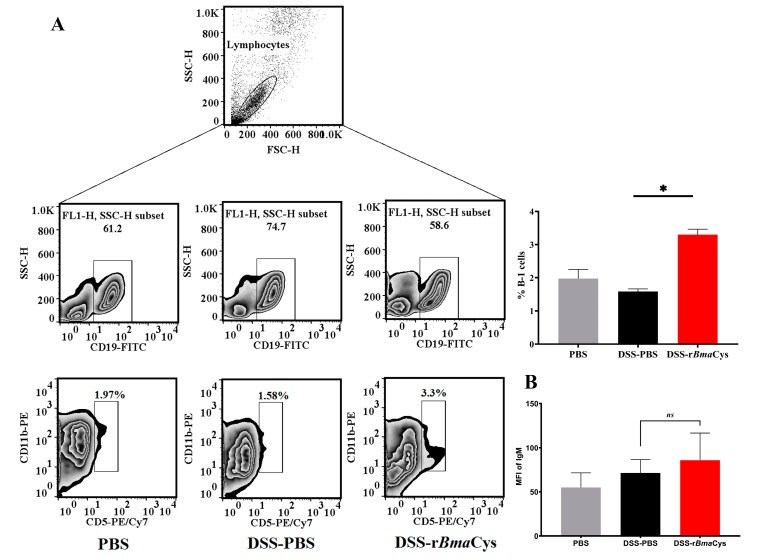
Frequencies of IgM^+^ peritoneal B1a cells. Peritoneal cells from each group of mice were plated for 24 h at 37 °C. Following incubation, the non-adherent cell population was collected, gated for CD19, and the percent of CD11b^+^CD5^+^ cells within the CD19^+^ population was identified as B1a cells. In addition, we also determined the MFI of IgM (using anti-IgM antibodies) in the B1a cells. Our results show that there was a significant increase in the percentage of B1a cells in the DSS-r*Bma*Cys-treated mice (**A**). Our analyses also showed an increase in the frequency of IgM producing B1a cells in the DSS-r*Bma*Cys-treated mice but was not significant compared to the DSS-PBS mice (**B**). Each bar represents mean ± S.D. * *P* = 0.006 compared to DSS-PBS group determined using one-way ANOVA followed by Tuckey’s posthoc test, *n* = 10 mice per group, except the DSS-PBS group, which had 8 mice. *ns* = not significant.

The percentage of B1a cells was significantly (*P* = 0.006) increased in the peritoneal cavity of DSS-r*Bma*Cys-treated mice (3.3%) compared to the DSS alone group of mice (1.58% Figure 6). The frequencies of IgM^+^ B1a cells also increased in the peritoneal cavity of DSS-r*Bma*Cys-treated mice (Σ IgM = 85.95) compared to the DSS alone group of mice (Σ IgM = 71.47; Figure 6).

## 3. Discussion

Results presented in this study show that treatment with r*Bma*Cys significantly reduced the clinical symptoms of colitis such as a reduction in the loss of body weight and development of hematochezia. Similarly, colon pathology was also minimal in r*Bma*Cys-treated mice despite DSS administration. This was associated with diminished inflammatory responses in the mucosa of r*Bma*Cys-treated mice that received DSS. Analysis of the immune responses in the colon, regional lymph nodes, and peritoneal spaces of r*Bma*Cys-treated mice showed significant increases in TNF-α and IL-6 producing peritoneal macrophages, Tregs, and IgM^+^B1a cells.

The clinical findings in this study confirmed the previous report [8] showing that the treatment with r*Bma*Cys ameliorated the clinical symptoms and colon pathology following administration of DSS in mice. One of the typical characteristics of DSS-induced colitis in mice is a substantial reduction in the size of the colon [8,12] by as much as half the normal size along with severe inflammation and edema in the mucosa. However, following treatment with r*Bma*Cys, the reduction in colon length was only 26%. Thus, r*Bma*Cys appears to have a clear effect on the tissue pathology reducing the damage induced by DSS to the colon. As reported previously [8,13], findings in this study also clearly show that *B. malayi* cystatin has potent immunomodulatory activity downregulating the pathology in the colon.

In colitis, there is diffuse inflammation and continuous infiltration of inflammatory cells into the mucosa [14]. The major cells that infiltrate the colon mucosa include neutrophils that can cause significant tissue damage by producing several toxic enzymes and proinflammatory mediators including cytokines and chemokines thereby promoting severe mucosal inflammation that further aggravates the colitis condition [15]. In this study, we did not analyze the inflammatory cells in the colon mucosa, but the mucosa was highly edematous with severe infiltration of cells in the control mice that received solely DSS. However, in mice treated with r*Bma*Cys, the inflammation appeared substantially reduced and there was minimal mucosal edema. These findings suggested that r*Bma*Cys-treatment may have prevented the migration and accumulation of inflammatory cells in the colon tissue, which may have played a major role in alleviating the acute symptoms of DSS-induced colitis in the mice.

We next investigated the mechanisms of the diminished inflammatory changes in the colon tissue following r*Bma*Cys-treatment. IL-17 and TNF-α can promote leukocyte infiltration in the colon at the site of inflammation [16]. Thus, suppression of IL-17 has been shown to diminish the inflammation in the colon following induction of colitis [17], whereas, induction of IL-17 exacerbates colitis symptoms [18]. In this study, we found a decrease in the expression of both IL-17 and TNF-α in the colons of r*Bma*Cys-treated animals that were given DSS. Secondary cytokines such as TNF-α, IL-1β, and IL-6 are known to increase the presence of inflammatory cells in the colon tissue in response to primary Th1/Th17 immune responses in inflammatory bowel disease (IBD) thereby synergistically drawing the inflammatory milieu [19]. In the present study, there was a significant decrease in the expression of IL-6 following r*Bma*Cys-administration in DSS-induced colitis mice.

Innate lymphoid cells (ILCs) are known to be important for intestinal homeostasis and can lead to the onset of IBD [20]. ILC2 cells, which are homologous to Th-2 cells, express the transcription factor GATA-3, and ILC3 cells, which are homologous to Th17 cells, express the transcription factor ROR-γ [21,22]. Treatment with r*Bma*Cys in DSS-induced colitis mice slightly decreased the levels of GATA-3 in the colon cells, however, there was a significant decrease in the expression of ROR-γ in the colon cells after r*Bma*Cys-treatment. In addition, we observed down-regulation in the expression of the transcription factors TBX21/T-bet in the colon cells of r*Bma*Cys-treated colitis mice. Previous studies in T-bet deficient mice showed that administration of DSS induced only milder colitis symptoms compared to wild-type mice [23]. These T-bet deficient mice also expressed lesser IFN-γ and IL-17 in their colon tissue and developed a Th2-type intestinal immune response [23]. Further studies are needed to understand the mechanism of r*Bma*Cys-induced regulation on T-bet expression in the colon and the maintenance of mucosal homeostasis.

Tregs are known to be a crucial player in providing protection from IBD in both humans and mouse models [24,25]. In the present study, r*Bma*Cys-treated colitis mice had elevated percentages of CD4^+^IL-10^+^ Tregs in LPMCs and CD4^+^ Tregs in MLNs. Similar increases in IL-10^+^Tregs have been reported by other groups also, where they demonstrated the therapeutic potential of cystatins from other helminth parasites in colitis [9,10,11,26]. IL-10 plays a major role in the cystatin-induced colitis and other immune-mediated disorders [7,26]. Blocking of IL-10 reversed the therapeutic effects of *A. vitae* cystatin suggesting that IL-10 play a major role in the cystatin-induced reversal of symptoms [26]. In our studies, we demonstrated that Tregs are a source of the IL-10 in r*Bma*Cys-treated mice. In addition to Tregs, macrophages are also important contributors of IL-10 in cystatin-mediated amelioration of autoimmune and allergic conditions [7,10,26]. Helminth-derived products have been shown to induce macrophage polarization thereby restricting the gut-inflammation in mouse models [27]. *S. mansoni* worms or its products can induce M2-phenotype in peritoneal macrophages [28,29] and can ameliorate the DSS-induced colitis symptoms in recepient mice [10,30]. Similarly, a single adoptive transfer of *A. viteae* cystatin-induced regulatory macrophages. Results from our study show that *rBmaCys*-treatment reduced the release of pro-inflammatory NO (nitric oxide) and enhanced the anti-inflammatory arginase activity and IL-10 expression in the peritoneal macrophages. Thus, similar to *S. mansoni* cystatin, the r*Bma*Cys protein also appears to have a direct effect on activating peritoneal macrophages and promoting an anti-inflammatory milieu via IL-10 production in the mucosa.

A role for IL-10-producing regulatory B1 cells and B cells in the suppression of autoimmune and inflammatory conditions is already established [31,32,33,34,35]. Adoptive transfer of IL-10-secreting B cells inhibited the onset of chronic collagen-induced arthritis in mice, suggesting a major role for regulatory B cells in autoimmune diseases [32]. Similarly, recent studies show that filarial antigens can stimulate the production of IL-10 and antigen-specific IgM antibodies from peritoneal B1a cells suggesting that the parasite may regulate the immune response in the host by manipulating regulatory B cells [29,36,37]. Very few attempts were made to identify the parasite antigens with the ability to stimulate regulatory B cells. ES-62 antigen isolated from the rodent filarial parasite, *A. viteae* has the ability to promote the expansion of IL-10-producing B-cells and this was associated with a reduction in the symptoms of arthritis [38,39]. However, no previous data is available on the potential role of filarial cystatin on B-cells. Our current data suggest that r*Bma*Cys can promote increased numbers of IgM^+^B1a cells in mice. However, we were not able to see any increase in the frequencies of IL-10^+^B1a cells. Studies have demonstrated the potential therapeutic role of IgM, IL-10, and adenosine-producing B-1a cells during intestinal inflammation [37]. Similarly, mice with increased frequencies of IgM^+^B1a cells are less prone to develop colitis [40]. Studies by Kiyohara et al. [41] show that IgM^+^B cells are absolutely necessary for protecting the mice from colitis symptoms. When the frequencies of IgM^+^B cells were reduced in both colon and peritoneum, the mice showed severe colitis symptoms suggesting a central role for B1 cells in suppressing colitic inflammation [41]. Similarly, patients with Crohn’s disease show remission when the frequencies of intestinal IgM and CR2/CD21 positive B cells were increased [42]. In this study, we did not identify the molecular mechanism by which r*Bm*Cys promotes an increased number of regulatory T cells and B1 cells. Nevertheless, increases in the numbers of IgM^+^ B1a cells observed in our studies following r*Bma*Cys-treatment correlated with the diminished inflammatory responses in the colon.

In conclusion, our findings demonstrated that *B. malayi* derived Cystatin (r*Bma*Cys), can alleviate the symptoms and pathology of colitis by driving the localized Th2 immune responses and upregulating IL-10 production by Tregs, and increase in innate-immune cells including IgM^+^B1 cells. Further studies are needed to define the mechanism of action of cystatin and the various immune receptors involved in driving these immunomodulatory effects in the colon tissue.

## 4. Materials and Methods

### 4.1. Expression and Purification of rBmaCys

The *bmacys* gene (NCBI Accession #XP_001902777) was synthesized at Thermo Fisher Scientific (Rockford, IL, USA) and was delivered in pET100 plasmid vector. A plasmid containing *bmacys* gene was transformed into NiCo21(DE3) expression systems of *Escherichia coli* (New England Biolabs, Ipswich, MA, USA). The recombinant *B. malayi* Cystatin (r*Bma*Cys) was expressed and purified as described previously [8]. Endotoxin from the recombinant protein was removed using ToxinEraser endotoxin removal kit (GenScript, Piscataway, NJ, USA). Endotoxin level in the final protein preparation was <3 EU/mg.

### 4.2. Animals

Six to eight-weeks-old, Balb/c male and female mice were purchased from Taconic Biosciences, Inc. (Hudson, NY, USA). The care and use of animals in this study were reviewed and approved by the IACUC Committee of College of Medicine, the University of Illinois at Rockford (BRC protocol # 1397385-3, 16 May 2019).

### 4.3. DSS-Induced Colitis Model

To induce the colitis, mice were given 5% (*w*/*v*) dextran sulfate sodium (DSS; 50,000 kDa; MP Biomedicals, Solon, OH, USA) ad libitum in drinking water for 7 days [43]. Control mice were given normal drinking water. Thirty-four Balb/c mice (17 male and 17 female) were divided into 4 groups. Group 1 (*n* = 10; five male and five female) mice received DSS in drinking water (DSS alone group); Group 2 (*n* = 10; five male and five female) mice received DSS in drinking water and 25 µg of r*Bma*Cys [8] intraperitoneally on days 4, 5, and 6 after starting the DSS administration (DSS/r*Bma*Cys-treated group); Group 3 (*n* = 4; two male and two female) mice received 25 µg of r*Bma*Cys intraperitoneally at the same time as in group 3 (r*Bma*Cys control) and Group 4 (*n* = 10; five male and five female) mice remained as control with no treatments (Control). The dose of r*Bma*Cys was as per the previous study [8]. All mice were monitored daily for clinical parameters such as body weight, stool consistency, blood in the stools, and diarrhea.

### 4.4. Evaluation of Colitis in the Mice Treated with rBmaCys

After euthanizing the mice, the colon was removed, and the length of the colon was measured by placing the colon right next to the markings on the ruler. Samples of the colon tissue from each mouse were then fixed in 10% buffered formalin for 24 h at room temperature. Fixed tissue was then processed for paraffin embedding at the pathology laboratory at the Mercyhealth Hospital (Rockford, IL, USA). Sections of the colon tissue (5–7 µm) was prepared and stained with hematoxylin and eosin (H&E). Histopathological changes in the tissue were scored under a light microscope using parameters described previously [8]. Samples of the colon tissue were also processed for immunohistochemical staining. Briefly, small pieces of the colon tissue were frozen in OCT compound and 10 µm sections were cut in a cryostat (Leica CM1860 UV, Leica Biosystems Inc, Lincolnshire, IL, USA). The sections were then fixed, blocked, and stained with monoclonal anti-mouse CD4 (FITC) and FoxP3 (APC) antibodies (Thermo Fisher Scientific, Rockford, IL, USA) to determine the distribution of Tregs in the colon tissue [44]. Total CD4^+^FoxP3^+^ cells in four different microscopic fields at 200× magnification were counted in the stained section of the colon tissue from each mouse. The values presented are the mean of the sum of total CD4^+^FoxP3^+^ cells counted from 10 mice from each group except the DSS-PBS group, which had only 8 animals.

### 4.5. qPCR Analysis to Determine Relative Cytokine mRNA Expression in the Colon Tissue and in Peritoneal Macrophages

To obtain enough cells from the colon tissue samples and to perform all the analysis, we pooled the colon tissue samples from five (5) male mice into one and the colon tissue samples from five (5) female mice into one for each group. Thus, we had only two samples (*n* = 2) of colon cells from each group of animals. The colon tissue was treated with 1 mL Trizol reagent (Sigma Aldrich, St. Louis, MO, USA) and total RNA was isolated using Direct-zol RNA miniprep plus kit (Zymo research Corp, Irvine, CA, USA). One microgram of this isolated RNA was reverse transcribed to cDNA using a Readyscript cDNA synthesis kit (Sigma Aldrich, St. Louis, MO, USA).

Before collecting the colon tissue from the euthanized mice, we washed the peritoneal cavity of each mouse with 1 mL sterile saline solution and the peritoneal fluid was carefully collected into a sterile tube. Peritoneal macrophages were collected from each mouse by incubating the peritoneal fluid in 6 well culture plates at 37 °C and 5% CO_2_ for 4 h. After removing the non-adherent population (processed separately for B1 cells flow cytometry), the plate was washed three times with sterile RPMI medium. The cells were then lifted from the plates, counted and plated again on a 24-well culture plate at a concentration of 1 × 10^6^ cells/well. The cells were then incubated for 24 h in RPMI 1640 media supplemented with 10% fetal bovine serum. Both culture supernatant and cells were collected separately. Trizol reagent was added to the cells and total RNA was isolated from the adherent cells using Direct-zol RNA miniprep plus kit (Zymo research Corp, Irvine, CA, USA). Isolated RNA (400 µg) was used as a template to perform reverse transcriptase PCR reaction using Readyscript cDNA synthesis kit.

IL-6, IL-10, RORγ, TNF-α, GATA-3, TBX21, and IL-17a genes were qPCR amplified, from the colon tissue cDNA prepared above. Similarly, TNF-α, IL-6, and IL-10 genes were qPCR amplified from the peritoneal macrophages, using the pre-optimized TaqMan Gene expression Assays and TaqMan^®^ multiplex master mix (Thermo Fisher Scientific, Rockford, IL, USA). GAPDH was included in the assay as a housekeeping gene. PCR amplification was carried out on QuantStudio 6 Flex Real-Time PCR system and the amplification data was analyzed using the QuantStudio Real-Time PCR software version 1.1 (Thermo Fisher Scientific, Rockford, IL, USA).

### 4.6. Levels of Nitric Oxide in the Culture Supernatants of Peritoneal Macrophages

The levels of nitric oxide in the culture supernatants from the peritoneal macrophage cultured above were determined using Griess reagent (Sigma-Aldrich, St. Louis, MO, USA). After removing the culture supernatant, the cell pellets were analyzed for the levels of arginase 1 activity using an Arginase activity assay kit (Sigma-Aldrich, St. Louis, MO, USA).

### 4.7. Flow Cytometric Analysis of Treg and B-1 Cells

#### 4.7.1. Analysis of the LPMCs

LPMCs were isolated from the colon tissue as described previously [45]. Before staining, the isolated LPMCs were blocked with mouse Fc blocker (BD Biosciences, San Jose, CA, USA) and incubated with a cocktail of anti-mouse CD3 (PE; clone 17A2), anti-mouse CD4 (PerCP; clone GK1.5) and anti-mouse CD25 (PE/Cy7; clone PC61.5) antibodies (Thermo Fisher Scientific, Rockford, IL, USA) for 1 h at 4 °C in the dark. Cells were then washed with staining buffer (2% FBS + 0.1% sodium azide), fixed in 4% paraformaldehyde (Biolegend, San Diego, CA, USA), and permeabilized for intracellular staining by incubating twice in the Intracellular Staining Perm Wash Buffer (Biolegend, San Diego, CA, USA) for 10 min. After permeabilization, cells were incubated with a cocktail of anti-mouse FoxP3 (APC; clone FJK-16S) and anti-mouse IL-10 (FITC; clone JES5-16E3) antibodies in a mouse Fc blocker solution. Data were acquired on a BD FACS Melody flow cytometer and analyzed using FlowJo v10.4.2 software (FlowJo, LLC, Ashland, OR, USA).

#### 4.7.2. Analysis of Spleens and MLNs

The spleen was aseptically removed from each mouse, washed twice in RPMI complete medium and the single cell suspension was prepared. After lysing the erythrocytes using ACK lysis buffer (Thermo Fisher Scientific, Rockford, IL, USA), cells were washed twice and counted. 2 × 10^6^ splenocytes were then added to the wells of a 24-well plate coated with anti-mouse CD3. Anti-mouse CD28 antibody (BD Biosciences, San Diego, CA, USA; 1ug/mL/well) was then added to the wells and incubated for 72 h.

Single cell suspension of MLNs collected from each group of mice was prepared. Both splenocytes and MLNs were then stained with the same set of antibodies described above for the LPMCs. Data were acquired and analyzed in a flow cytometer.

#### 4.7.3. Analysis of Peritoneal B1 Cells

The non-adherent cells in the peritoneal fluid collected after removing the adherent cells were then washed by centrifugation. Cells were fixed in 4% paraformaldehyde and stained with anti-mouse CD19 (FITC; clone 6D5), CD11b (PE; clone M1/70) and CD5 (PE/Cya7; clone 53–7.3) antibodies. After permeabilizing the cells, intracellular IgM (clone 11/41) was detected using respective antibodies labeled with APC and analyzed in a flow cytometer.

### 4.8. Statistical Analysis

Statistical analysis was performed using GraphPad Prism (v7.0; GraphPad Software, La Jolla, CA, USA). Significance between the groups was assessed using the non-parametric Kruskal Wallis or Students *t* test and parametric one-way ANOVA test based on the normality assumptions of the data. Multivariate analysis was carried out using suitable post-hoc test. *P* ≤ 0.05 was statistically significant with a 95% confidence interval.

## Figures and Tables

**Table 1:** Histopathological scoring of colon sections.

Groups	Histopathological Score
	(% Change with Respect to DSS-PBS Group)
PBS	No change
DSS-PBS	100
DSS- r*Bma*Cys	75.4
r*Bma*Cys only	No Change
Sections of colon tissues from each animal were fixed, paraffin-embedded and H&E stained. Each section was scored for pre-defined histopathological gradations by a sample blinded independent histopathologist. *n* = 8–10 mice per group. The variations within the groups were not-significant as per the One-Way ANOVA followed by *Tukey’s* post-hoc test.	
**Scoring Parameters**	**Score**
**Inflammation extent**	
None	0
Mucosa	1
Mucosa and submucosa	2
Transmural	3
**Inflammation severity**	
None	0
Mild	1
Moderate	2
Severe	3
**Crypt damage**	
None	0
Superficial 1/3 damage	1
Superficial 2/3 damage	2
Patchy crypt lost; surface epithelium present	3
Crypts & surface epithelium lost	4
**Colon wall thickening**	
None	0
Mild	1
Moderate	2
Marked increase	3
**Leukocyte infiltration**	
Normal	0
Slight increase	1
Moderate increase	2
Marked increase	3
**Lamina propria mononuclear cells**	
Normal	0
Slight increase	1
Moderate increase	2
Marked increase	3
